# Efficacy of Yokukansankachimpihange on sleep disturbance in Parkinson's disease: A study protocol of a randomized, double blind, placebo-controlled pilot trial

**DOI:** 10.1097/MD.0000000000011298

**Published:** 2018-06-29

**Authors:** Jung-Hee Jang, JuAh Lee, Inchul Jung, Horyong Yoo

**Affiliations:** a Department of Neurologic Disorders & Aging Brain Constitution, Dunsan Korean Medicine Hospital, Daejeon University, Daedeok-daero, Seo-gu, Daejeon, South Korea; b Department of Korean Internal Medicine, College of Korean Medicine, Gachon University, Keunumul-Ro, Jung-Gu, Incheon, Republic of Korea; c Department of Korean Neuropsychology, Dunsan Korean Medicine Hospital, Daejeon University, 75, Daedeok-daero, Seo-gu, Daejeon, South Korea.

**Keywords:** functional near-infrared spectroscopy, Parkinson's disease, polysomnography, protocol, sleep disturbance, Yokukansankachimpihange

## Abstract

**Introduction:**

Parkinson's disease (PD) is a progressive neurodegenerative disorder that includes motor and nonmotor symptoms. Sleep disturbance is known to decrease the quality of life in patients with PD, and there are limitations to the pharmacotherapy currently in use. Therefore, complementary treatment therapies are required to address these limitations. The traditional herbal medicines Yokukansan (YKS) and Yokukansankachimpihange (YKSCH) have been used to treat insomnia and night crying in children, suggesting their effectiveness against sleep disturbance in patients with PD. We will evaluate whether YKSCH improves sleep disturbance in PD and will identify YKS-related changes in hemodynamic parameters, and neurotransmitter and hormone levels in patients with PD experiencing sleep disturbance.

**Methods:**

We will conduct a randomized, double-blinded, placebo-controlled parallel trial in 34 patients with PD and sleep disturbance, randomly allocating the patients to either placebo-control (n = 17) or treatment groups (n = 17). The total study period will be 16 weeks; administration of YKSCH or placebo, as intervention, will be performed for a 12-week period, and follow-up will be performed over a 4-week period. All subjects will undergo conventional treatment, and be required to maintain a regular medication schedule throughout the study period. The primary outcome measure will be the Scales for Outcomes in PD-Sleep Scale score, and the secondary outcome measures will be polysomnography results, findings from instruments related to sleep disorders, neurotransmitter and hormone levels, and hemodynamic changes in the brain cortex.

**Discussion and conclusions:**

This trial will evaluate the effectiveness and safety of YKSCH for sleep improvement in PD with sleep disturbance, and investigate the underlying mechanism of action. We expect improvement in the scores for subjective and objective sleep scales, hemodynamic changes in prefrontal cortical activity, and changes in neurotransmitter and hormone levels. The findings will provide insight into the mechanism underlying the therapeutic effect of YKSCH in PD, and lay the foundation for further studies on whether YKSCH improves sleep disturbance in PD.

**Trial registration number:**

Clinical Research Information Service (KCT0002869).

## 1 Introduction

Parkinson's disease (PD) is the second most common progressive neurodegenerative disease and has multiple symptoms.^[1]^ Although PD is clinically defined by its motor features,^[1]^ more than 90% of the patients with PD experience decreased quality of life due to nonmotor symptoms, and the interest in addressing non-motor PD symptoms is increasing.^[2]^ The standard therapy for PD is levodopa, monoamine oxidase (L-DOPA) administration, but it causes several side effects. Most significantly, the long-term use of L-DOPA and dopamine agonists induces motor complications, psychiatric problems, and sleep disturbance.^[3]^ Among the several nonmotor symptoms of PD, sleep disorder is known to be multifactorial,^[4]^ and associations between sleep disturbances and other non-motor symptoms have been reported.^[5,6]^ In addition, studies have demonstrated circadian disturbances in patients with PD exhibiting comorbid sleep disorders, which seem related to changes in cortisol and melatonin hormone levels.^[7,8]^ Recently, doxepin administration and microsubthalamotomy have been reported to be effective therapies against sleep disturbance therapy in PD.^[9,10]^

Yokukansan (YKS) is a traditional medicine that is used for insomnia, night crying in children, and neurosis.^[11]^ Administration of YKS for 8 weeks to patients with dementia is beneficial in terms of sleep disturbance metrics, such as improvement of the sleep disorder inventory score and objective actigraphic evaluations.^[12]^ Mechanistic studies in mice suggest that YKS operates by enhancing pentobarbital-induced sleep involving γ-aminobutyric acid type A (GABA_A_) receptors,^[13]^ and inducing a sleep-promoting effect by causing a decrease in body temperature.^[14]^ However, previous studies on the effect of YKS on sleep disturbance are limited, and mostly focus on neuropsychiatric disorders. Recently, a clinical trial of YKS in PD reported improvement of neuropsychiatric symptoms, such as hallucinations, anxiety, and apathy, using an established Neuropsychiatric Index (NPI).^[3,15]^ A therapeutic role for YKS against psychiatric symptoms is in line with previous findings; YKS attenuated hallucination-like behaviors, such as head-twitch response induced by 2,5-dimethoxy-4iodoamphetamine, and down-regulation of serotonin 5-HT_2A_ receptor density in the prefrontal cortex (PFC) of isolation-stressed mice.^[16]^ In another study, repeated administration of YKS in a restraint-stress rat model led to decreased anxiety-like behavior and stress-induced c-Fos expression in the medial PFC.^[17]^ In addition, anxiolytic effects of YKS were found to involve serotoninergic and dopaminergic transmission.^[18]^

A variant of YKS, called Yokukansankachimpihange (YKSCH), which is a combination of 2 herbs (*Citrus unshiu* peel [Chimpi] and *Pinellia* sp. tuber [Hange]) with YKS,^[19]^ was found to be effective in improving sleep by increasing total sleep time and sleep efficiency of normal healthy adult subjects.^[20]^ In a mouse model of aggressive behavior caused by zinc deficiency, administration of drinking water containing YKSCH decreases aggression by suppressing excessive glutamatergic neuronal activity in the hippocampus.^[19]^ However, only a few studies have evaluated the effects of YKSCH on the improvement of sleep disturbance in PD.

Here, were report the plans for and design of a study that will evaluate the effects of YKSCH on sleep improvement in patients with PD experiencing sleep disturbance. This study will be a prospective, randomized, double-blinded, placebo-controlled, parallel-group pilot trial. The subjects will be patients with PD who are experiencing sleep disturbances, and these subjects will be randomly assigned to a treatment group or control group at a 1:1 ratio. The intervention will be YKSCH administration in the treatment group and placebo administration in the control group. Subjective and objective assessments of sleep improvement will be performed in this study using a questionnaire survey instrument, polysomnography (PSG), and sleep-related hormone measurements. To elucidate the mechanism underlying the effects of YKSCH in PD, hemodynamic changes in the frontal lobe will be measured using functional near-infrared spectroscopy (fNIRS), and the levels of neurotransmitters (NTs) related to PD will also be evaluated.

## 2 Methods and design

### 2.1 Study design and setting

This study will be a randomized, placebo-controlled trial to identify the efficacy of YKSCH in the treatment of sleep disturbance in PD. During the clinical trial period, all the subjects will be allowed to undergo any combination therapy, such as conventional medicine treatment, non-pharmacotherapy, and exercise therapy, except herbal medicine treatment. In combination therapy, the dosage of anti-PD conventional medication will have to have been stable for at least 1 month prior to enrollment. The primary outcome measure will be the Scales for Outcomes in PD-Sleep Scale (SCOPA-S) score, and the secondary outcome measures will be PSG findings; results of questionnaire survey on non-motor symptoms in PD; changes in NTs and hormone levels, hemodynamic changes in the PFC, as measured by fNIRS in patients with PD. Descriptions of selection of subjects, clinical outcomes, and safety assessment are presented in Table 1. The study setting and site of data collection is the Clinical Trial Center at Dunsan Korean Medical Hospital of Daejeon University in South Korea.

**Table 1:**
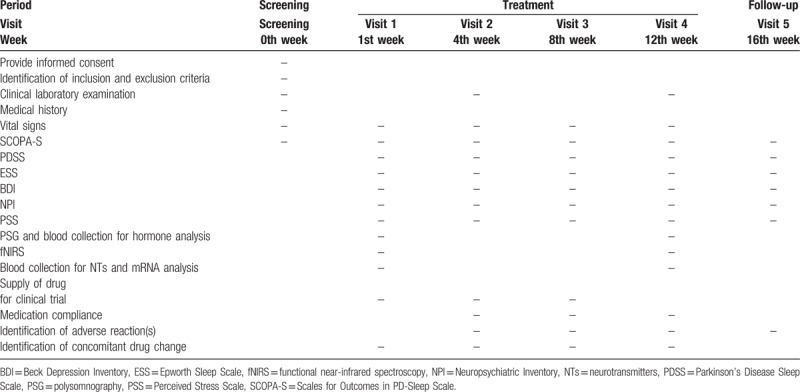
Schedule for selection of subjects, clinical outcomes, and safety assessment.

### 2.2 Participants and recruitment

In total, 34 subjects with PD experiencing sleep disturbance will be recruited through posters placed in the community and hospital, local newspapers, and subway advertisements, from July to October 2018. Subjects who are eligible according to inclusion criteria will provide written informed consent after a consultation, and receive information regarding the clinical study from the coordinator. They will then be randomly allocated to the treatment or control groups.

### 2.3 Eligibility criteria

#### 2.3.1 Inclusion criteria

A diagnosis of PD made by a neurologist; SCOPA -night score ≧7; Hoehn and Yahr Scale stage 1–4; and provision of informed consent.

#### 2.3.2 Exclusion criteria

Patients with Alzheimer's dementia, vascular dementia, Huntington's disease, amyotrophic lateral sclerosis, or hydrocephalus; gait disturbance caused by stroke, brain tumor, or other brain diseases; a diagnosis of gastrointestinal disease, endocrine disease, or cardiovascular disease not controlled by diet or medication; clinical laboratory abnormality, such as serum creatinine ≥97 mmol/L, alanine amino transferase ≥ 40 U/L, aspartate amino transferase ≥ 40 U/L; unstable medical condition, as decided by the research clinician; administration of herbal medication for PD within the last 4 weeks; and inability to undergo fNIRS. Furthermore, patients may be considered ineligible to participate in this clinical study for other reasons, according to the coordinator's discretion

### 2.4 Randomization and blinding

The allocation to placebo control or YKSCH treatment group of subjects meeting the eligibility criteria will be performed by an independent statistician via computer-generated block randomization. For assessing secondary outcomes, PSG and hormone analysis will be performed for some of the subjects from the treatment and control groups at a 1:1 ratio; the subjects will be selected by an independent statistician, using systematic sampling. Each subject will receive an identification code enclosed in an opaque envelope. The identification code shall indicate whether PSG and hormone analysis will be performed. The subjects, researchers, and assessors collecting the data will be blind to group assignment, as this study is designed to be double blind. If unblinding is required, such as occurrence of medical emergency, the principal investigator decides to remove the blindness through the researchers’ meeting. In this case, the institutional review board (IRB) should be informed of the unblinding and explain the medical emergency in detail within 24 hours enrollment of subjects, allocation sequence, and assignment of subjects will be the responsibility of the Clinical Trial Center at Dunsan Korean Medical Hospital of Daejeon University.

### 2.5 Interventions

Both groups will receive conventional anti-PD medicines, including L-DOPA, dopamine agonists, catechol-*O*-methyl transferase (COMT) inhibitor, and monoamine oxidase (MAO) inhibitor, and will be prohibited from undergoing herbal medicine treatment. We will perform all examinations and assessments in the dopaminergic “on” state (the same time in each patient, after administering the last dose of conventional medication). A constant dose of conventional medication will be maintained during treatment and the follow-up period. If receiving neuropsychiatric drugs, such as sedatives, hypnotics, anxiolytics, selective serotonin uptake inhibitor, and sleep stabilizers, a stable dose of these drugs will have to have been maintained for at least 1 month prior to enrollment. Both YKSCH and placebo drugs will be packed in opaque sachets in granular form to blind researchers and subjects, and will be administered orally 3 times daily. Criteria for discontinuing allocated interventions for subjects are: withdrawal of the clinical trial participation agreement by subjects or their legal representatives, violation of the selection/exclusion criteria during clinical trial, occurrence of adverse events, and less than 70% compliance with medication criteria. To improve adherence to interventions, the *clinical research coordinator will assess medication criteria adherence (number of medicines received/number of medicines to be received)* every time the subjects visit the clinic.

#### 2.5.1 Treatment group: YKSCH

Nine dried medicinal herbs constitute YKSCH: *Atractylodis rhizoma*, *Poria sclerotium*, *Cnidium rhizome*, *Uncaria* sp. hook, Japanese Angelica root, *Bupleurum* sp. root, *Glycyrrhiza* sp., *Citrus* sp., and *Pinellia ternata*. The treatment group will receive YKSCH. The formula will be supplied by the manufacturer Kyungjin Pharmaceutical Co., Ltd. (Icheon, Republic of Korea), and be manufactured in compliance with Good Manufacturing Practice under Ministry of Food and Drug Safety guidelines. The final form of YKSCH is granular.

#### 2.5.2 Control group: placebo

The control group will be administered the placebo drug. The placebo drug will be manufactured using the same process as YKSCH, by the same manufacturer; it will be made of lactose, starch, and caramel coloring.

### 2.6 Outcome measures

Any outcome assessments without PSG and hormone analysis will be performed in the dopaminergic “on” state at the same time for each subject, after administering the last dose of conventional anti-PD medicine.

**Figure 1:**
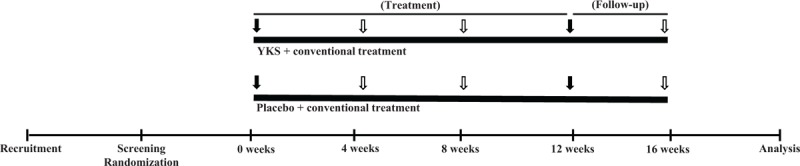
Schedule of YKSCH or placebo treatment and collection of outcome measurements. The treatment or control groups will be administered YKSCH or placebo drug, respectively. The subjects will receive YKSCH or placebo for 12 weeks, and be followed-up for 4 weeks. Administration of SCOPA-S, to assess primary outcome; PSG; collection of blood samples for analysis of NT and hormone levels; fNIRS measurement will be performed at 0 and 12 weeks (indicated by black arrows). Questionnaire surveys related to sleep disturbance will be conducted at 0, 4, 8, 12, and 16 weeks (indicated by black and hollow arrows). fNIRS = functional near-infrared spectroscopy, NT = neurotransmitter, PSG = polysomnography, SCOPA-S = Scales for Outcomes in PD-Sleep Scale, YKSCH = Yokukansankachimpihange.

The schedule for outcome measurements is presented in Figure 1.

#### 2.6.1 Primary outcomes

##### 2.6.1.1 Scales for outcomes in PD-sleep scale

The score of SCOPA-S, which is a questionnaire survey assessing problems in both night- and day-time sleep, and the measurement of PD^[4,9,21]^ the severity will be the primary outcomes measured in this study. The survey will be conducted at baseline (visit 1) and 12 weeks after treatment (visit 4) for both groups.

#### 2.6.2 Secondary outcomes

The secondary outcome measures include findings from 5 instruments evaluating sleep disorders, PSG and sleep-related hormone analysis, changes in the levels of NTs associated with PD, and hemodynamic changes in the PFC during verbal fluency task of subjects.

#### 2.6.3 Instruments to assess sleep disturbance in PD

Parkinson's Disease Sleep Scale (PDSS), Epworth Sleep Scale (ESS), NPI, Beck Depression Inventory (BDI), and Perceived Stress Scale (PSS) scores will be assessed at visits 1–5 for both groups. The PDSS and ESS are valid tools for evaluating sleep disturbance in PD^[22]^ and measuring day-time sleepiness,^[23]^ respectively. For assessing several symptoms related to sleep disturbance, NPI,^[24]^ BDI,^[6]^ and PSS^[25]^ scores will be assessed as secondary outcomes. The NPI is considered a valid instrument to evaluate behavioral and psychological symptoms, including sleep disorder.^[24]^ The BDI will be used to assess depression, one of the leading causes of insomnia.^[6]^ Stress is also known to be associated with sleep quality, and the PSS assesses subjective appraisals of stressful situations.^[25]^

#### 2.6.4 Polysomnography and hormone analysis

For objective sleep assessment, total recording time, sleep period, waking after sleep onset, total sleep time, sleep onset, sleep efficiency, number of awakenings, sleep latency to N1, sleep latency to N2, sleep latency to N3 (slow wave sleep), and stage R latency from sleep onset will be measured using PSG (Embletta MPR PG, Natus Medical Incorporated, Pleasanton, CA, USA). The subjects will be examined using in-laboratory PSG for one night. The PSG assessment will be set up and performed by a trained sleep technologist, using an Embletta MPR.^[26]^ This device consists of multiple channels for electroencephalography, electrocardiography, electrooculography, airflow, etc. Recordings from the electroencephalogram channels (C3-M2, C4-M1, O1-M2, O2-M1, F3-M2, and F4-M2) in the frontal, central, and occipital regions of the head, electrooculography channels (LOC-M2 and ROC-M1), and electromyography channel (EMG1-EMG2) in both legs will be acquired for each subject. Nasal and oral airflow, recorded using oral/nasal thermistor airflow and pressure nasal cannula, will be measured. In addition, oxygen saturation, using pulse oximetry; movements of the chest and abdomen; behavior during sleep, using video recording; change of body position, such as supine, left, right, prone, and upright, and snoring; and pulse rate will be recorded. Scoring of the sleep stages will be performed according to the American Academy of Sleep Medicine rules.^[27]^ Blood sampling, for measurement of cortisol and melatonin levels, will be conducted every 4 hours, over 24 hours, via venous catheter. Hormone levels will be examined using a melatonin enzyme-linked immunosorbent assay (ELISA) kit (ab213978; Abcam, Cambridge, MA) and a cortisol parameter assay kit (KGE008B, R&D systems, Minneapolis, MN).

#### 2.6.5 Molecular analysis: plasma and serum levels of neurotransmitters

Investigation of changes in the plasma and serum NT levels may help us to understand the mechanism of action of YKSCH in patients with PD experiencing sleep disturbance. The serum levels of the PD-related NTs, dopamine, GABA, glutamate, norepinephrine, epinephrine, and serotonin, will be analyzed.^[28]^ Blood collected from the patients in both groups, at baseline (visit 1) and after treatment (visit 4), will be immediately centrifuged at a temperature below 4°C. The obtained plasma and serum samples will be stored at a temperature below −80°C until further analysis. The samples will be analyzed by Seoul Clinical Laboratories in South Korea, a quality facility certified by the European Quality Assurance Agency. The levels of dopamine, epinephrine, and norepinephrine will be measured using a high-performance liquid chromatography-electrochemical detection system (Agilent Technologies, Santa Clara, CA). The glutamate and GABA levels will be measured using ion-exchange chromatography (Biochrom Ltd., Cambourne, Cambridge, UK). The serotonin levels will be measured using the ELISA Fast Track kit (Labor Diagnostika Nord, Nordhorn, Germany). For hormone analysis, the subjects will be asked to abstain from drinking, smoking, and consumption of caffeine, turkey meat, bananas, and tomatoes, beginning the day before the test, as these can affect sleep rhythm and melatonin secretion.^[8]^ The night before hormone analysis, the subjects will be instructed to turn off the lights and attempt to sleep at 10 pm, and the use of electronic devices, such as smart phones and televisions, will be prohibited on the day before the evaluation.

#### 2.6.6 Neuroimaging: functional near-infrared spectroscopy

The response in the frontal regions of the brain has previously been measured using fMRI during a verbal fluency task (VFT) in patients with chronic insomnia, and is known to be affected by sleep deprivation; in this study, decreased activation in the cortex was attenuated after effective treatment.^[29]^ Hemodynamic responses, measured using fNIRS, during a VFT showed hypoactivation in the PFC of patients with chronic insomnia.^[30]^ Therefore, our intention is to determine whether the concentrations of oxygenated hemoglobin in the PFC of patients with PD exhibiting sleep disorder is increased by YKSCH administration, using fNIRS during VFT. The test will be performed at baseline (visit 1) and after treatment (visit 4) in both groups. The subjects will perform a VFT in which they must speak as many items as possible within the given semantic categories. The measurements will be performed by an experienced user of the fNIRS system.

The noninvasive optical imaging, fNIRS system (NIR Scout 1624, NIRx Medical Technology, Berlin, Germany) is used to measure changes in the cerebral concentrations of oxyhemoglobin and deoxyhemoglobin.^[31]^ This system utilizes the transmission of near-infrared light at 2 wavelengths (760 and 850 nm), with a power of 5 mW per wavelength, and comprises 16 light-emitters and 16 photodetectors. The probes will be placed on the head of each subject, at a region corresponding to the PFC. Data collection and analysis will be performed using Nirs LAB version 201601 (NIRx Medizintechnik GmbH, Berlin, Germany).

Based on the difference in absorption spectra of the detected signal, assuming constant light scattering, changes in the concentrations of oxyhemoglobin and deoxyhemoglobin in the targeted PFC region will be measured using the modified Beer–Lambert law.^[32]^ The data for the measured oxyhemoglobin and deoxyhemoglobin concentrations will be exported to MATLAB (MATLAB and Statistics Toolbox Release 2012b, The Math Works, Inc., Natick, MA),^[32]^ calculating the mean oxyhemoglobin and deoxyhemoglobin concentrations for each patient.

### 2.7 Participant timeline

**Figure 2:**
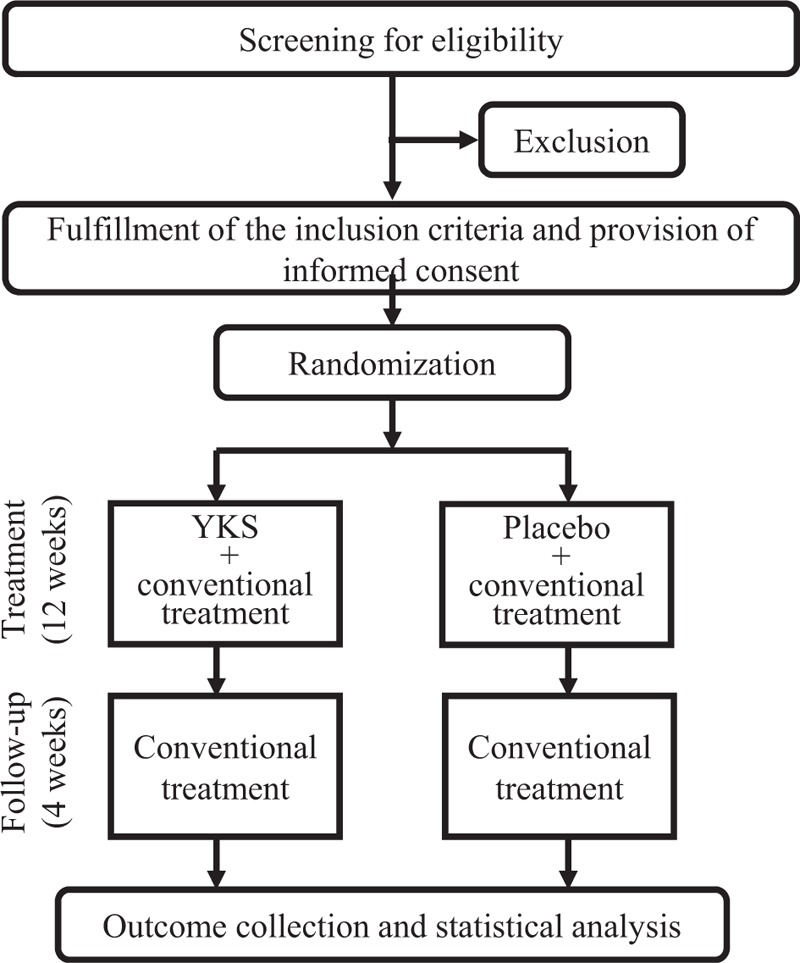
The study design flowchart showing details of the randomized controlled trial.

The total study duration will be 16 weeks, including a 12-week treatment period and a 4-week follow-up period. A flow chart depicting the study design is presented in Figure 2.

### 2.8 Safety assessment and adverse events

Safety assessment for all subjects will be performed via clinical laboratory examination at baseline, before drug administration; 4 weeks after drug administration (visit 2); and 12 weeks, after completing drug administrations (visit 4). Laboratory tests will include those for live rand renal function, which can possibly be damaged by herbal medicines,^[2]^ and serum potassium concentration because *Glycyrrhiza radix*, a component of YKSCH, can induce hypokalemia.^[3]^ Adverse events will be defined as undesirable and/or unexpected medical findings which did not occur before the start of this clinical trial, and will be recorded, regardless of their relation to YKSCH or the placebo drug. The severity of the adverse events will be evaluated based on the step-wise criteria established by the chief investigator, and reported to the IRB of Daejeon University. Following appropriate deliberation, the IRB will determine whether the trial should be discontinued. Causal link between the adverse events and the procedure will be investigated by the doctor-in-charge, and categorized into 6 stages based on the aforementioned evaluation criteria. In case of a severe adverse event, appropriate treatment and follow-up will be provided.

### 2.9 Statistical methods

#### 2.9.1 Sample size

Designed to study the effects of YKSCH in patients with PD experiencing sleep disturbance, this pilot study will use the SCOPA-S score as the primary outcome. A total of 34 subjects (17 in each group) will be needed, based on the effect size demonstrated in a previous study on the effect of doxepin against insomnia in patients with PD,^[9]^ allowing a dropout rate of 10%, a two-sided significance level of 5%, and 80% statistical power.

#### 2.9.2 Statistical analysis

Statistical and fNIRS data analyses will be performed by statisticians and a researcher from a different group. An intention-to-treat analysis will be performed in case of subjects lost to follow-up, and multiple imputations will be applied for the missing data. Statistical analysis will be performed using SPSS software for Windows, version 24.0, (Chicago, IL). The SCOPA-S score, which is the primary outcome, will be evaluated by calculating the difference between the mean scores before and after receiving YKSCH or placebo (for treatment or control groups, respectively). Independent two-sample *t*-test (or the Mann–Whitney *U* test for nonparametric samples) will then be performed to analyze the difference between the treatment and control groups. The results of the questionnaire survey, including PDSS, ESS, NPI, BDI, and PSS, scored at 0, 4, 8, and 12 weeks, respectively, and NT levels measured at 0 and 12 weeks will be analyzed using independent two-sample t-tests (or the Mann–Whitney *U* test for non-parametric samples). To compare sleep metrics obtained using PSG and measurement of hormone levels, pre- and post-YKSCH or placebo treatment at weeks 0 and 12, paired t-tests (or the Wilcoxon signed-rank test) will be used. The mean oxyhemoglobin and deoxyhemoglobin concentrations determined using fNIRS at 0 and 12 weeks will be analyzed using repeated-measures analysis of variance. Pearson's correlation coefficient will be used to assess the association among subscales of the questionnaire surveys, oxyhemoglobin and deoxyhemoglobin concentrations, and NT levels. The level of statistical significance will be set at *P < *.05, and 95% confidence intervals will be calculated. A statistician will be consulted incase the obtained data need to be calibrated.

### 2.10 Data management and monitoring

The *clinical research coordinator* will fill out the case report forms, which shall include the information required by the protocol. Subsequently, 2 research assistants will independently enter the data into Excel sheets, and the results will be compared once the trial has been completed. All documents, for instance, the case report forms, will be preserved in the confidential archives at Dunsan Korean Medicine Hospital for 3 years. Any information obtained from the clinical trial will not be released without the permission of the principal investigator. An independent monitoring committee, comprised of individuals not related to the research team for this study, will regularly monitor patient safety, investigate adverse events, and review quality control of the data. An interim analysis will be performed at the request of the monitoring committee and/or the IRB. The audit will be conducted 3 times during the trial by a professional company not related to the research team.

### 2.11 Ethical considerations, informed consent, and study registration

The protocol for this study has been approved by the Research Ethics Committee of Dunsan Korean Medicine Hospital, Daejeon University, which accepts responsibility for supervising all aspects of the study (DJDSKH-18-DR-09). The potential risks and benefits will be explained to all the study participants and their families. Patients who are willing to participate will be included in the study after the submission of a signed informed consent form. The study protocol (version 1.1, 2018-05-11) has been registered with the Clinical Research Information Service of the Republic of Korea (KCT0002869). If there are significant protocol changes, the research coordinator will request supplementation of the IRB and trial registries. With regard to the protection of personal information of the subjects, they will be needed to provide a signed personal information usage and consent form, and the data collected through the clinical study shall be retained by the research institute for 3 years from the date of request for archiving, in accordance with the domestic regulations.

## 3 Discussion

Sleep disturbance is one of the most clinically significant non-motor symptoms of PD, and encompasses many symptoms such as insomnia, daytime sleepiness, restless legs syndrome, and rapid eye movement sleep behavior disorder, causing many inconveniences and stress to patients and caregivers.^[9,33]^ The characteristics of sleep disorder in PD are multifactorial and multidimensional, with overlapping symptoms.^[4]^ Therefore, assessing sleep disturbance in PD using a single instrument is difficult. To conduct a subjective as well as objective assessment of sleep disturbance, questionnaires on sleep, PSG, and hormone (cortisol and melatonin) analysis will be employed in concert in this study.

Subjective instruments to assess sleep disturbance, such as SCOPA-S, PDSS, and ESS, will be used as outcome measures in this study. The SCOPA-S and PDSS are used for specific assessments of sleep disturbance in PD.^[22]^ The SCOPA-S measures both nocturnal disturbance and day-time sleepiness. The PDSS measures nocturnal disability, and helps to identify the potential causes of sleep disorder.^[21,23]^ It has been validated to reflect observations of the patients and caregivers,^[34]^ and its clinical usefulness has been demonstrated.^[35]^ The ESS is used to measure day-time sleepiness.^[23]^ In addition, NPI, BDI, and PSS, questionnaire surveys on neuropsychiatric problems and stresses correlated with sleep disorder, have been selected for assessment of secondary outcomes.

In a previous study using PSG for an objective assessment, increased sleep latency, reduced sleep efficiency, and reduced rapid eye movement sleep in patients with PD have been reported.^[7]^ In a previous study on the use of microsubthalamotomy to improve sleep disturbance in patients with PD, the PSG data evidenced increased total sleep time and sleep efficiency, and decreased wakefulness after sleep onset and arousal index.^[10]^ In previous studies on circadian rhythms, amplitude of the melatonin rhythm was significantly lower during excessive day-time sleepiness in PD,^[36]^ and serum cortisol and circulating melatonin levels in early PD were elevated and reduced, respectively.^[7]^ Therefore, this study will seek to investigate, through PSG, whether YKSCH improves sleep time and efficiency, while further investigating the effects of cortisol and melatonin on sleep rhythm.

In addition to investigating the mechanism of action of YKSCH on sleep improvement, this study will assess the hemodynamic changes in the frontal lobe during a VFT using fNIRS, and the changes in NT concentrations will also be investigated. In a previous study, prefrontal cortical activation during VFT, assessed using fNIRS, was found to be reduced in chronic insomnia disorder.^[30]^ The purpose of this study is to investigate the mechanism of action of YKSCH in patients with PD exhibiting sleep disorder, by confirming the hemodynamic changes in the frontal lobe caused by the administration of YKSCH.

Sleep disturbance in PD is known to contribute to and exacerbate a variety of other conditions such as mood disorders, primary sleep disorders, night-time motor symptoms, and side effects of PD therapy.^[5]^ There are several studies on the relationship of sleep disturbance with non-motor PD symptoms such as depression, fatigue, and autonomic symptoms,^[5,6]^ and a number of other neuropsychiatric symptoms.^[37]^ Among symptoms associated with sleep disturbance, YKS has recently been mainly reported by several studies as a remedy for behavioral and psychological symptoms of dementia, such as aggressiveness and hallucinations.^[11]^ Therefore, it is expected to be a complementary treatment for sleep disorders related to neuropsychological symptoms, as well as primary sleep disorder, in patients with PD.

This study has some limitations. The study is a small pilot clinical trial. However, this study may be the basis for future large-scaled study because there is no evidence that confirms the effect of YKS of YKSCH on sleep disturbance in PD. In addition, it may be a basis for clarifying the mechanism of action of YKSCH by confirming sleep related hormone changes, identifying hemodynamic changes using fNIRS and neurotransmitter changes after administration of YKSCH. An another limitation, the severity of PD symptoms is not homogenous in all subjects. The interval between taking a dose of conventional and psychologic medication may differ between subjects. However, in an effort to minimize this bias, we are attempting to standardize the interval between taking medication.

The treatment of sleep disorders in patients with PD primarily involves pharmacotherapy with non-benzodiazepine hypnotics, sedating antipsychotics, and/or benzodiazepines. However, this approach may lead to side effects, including tolerance and dependency, and cognitive impairment, with decreasing effectiveness.^[33]^ Therefore, a new approach is needed to overcome the limitations of current pharmacotherapy options. This study will be the first clinical trial to evaluate the subjective as well as objective effects of YKSCH in patients with PD exhibiting sleep disturbance and hemodynamic changes in the brain cortex. We expect to demonstrate improvement in sleep disturbance; changes in the prefrontal cortical activity, using fNIRS; and changes in the concentrations of PD-related NTs to obtain a deep insight into the mechanism underlying the effect of YKSCH in PD. The results from this clinical trial will lay the foundation for further studies to confirm the effectiveness of YKSCH and identify potential alternative therapies to improve sleep disturbance in patients with PD.

## Author contributions

**Conceptualization:** Jung-Hee Jang, Inchul Jung, Horyong Yoo.

**Methodology:** Jung-Hee Jang, Inchul Jung, Horyong Yoo.

**Supervision:** Horyong Yoo.

**Writing – original draft:** Jung-Hee Jang.

**Writing – review & editing:** JuAh Lee.
